# Exploring the Effects of Giraffe Skin Disease Limb Lesions on Locomotion

**DOI:** 10.1002/ece3.71774

**Published:** 2025-07-10

**Authors:** N. T. Dunham, L. M. Bernstein‐Kurtycz, J. Manzak, A. B. Muneza, M. B. Brown, J. Fennessy, P. M. Dennis, C. J. Kendall, K. E. Lukas

**Affiliations:** ^1^ Division of Conservation and Science Cleveland Metroparks Zoo Cleveland Ohio USA; ^2^ Department of Biology Case Western Reserve University Cleveland Ohio USA; ^3^ Little Rock Zoo Little Rock Arkansas USA; ^4^ North Carolina Zoo Asheboro North Carolina USA; ^5^ Giraffe Conservation Foundation Windhoek Namibia; ^6^ Department of Veterinary Preventive Medicine The Ohio State University Columbus Ohio USA; ^7^ The Peregrine Fund Boise Idaho USA; ^8^ Department of Applied Ecology North Carolina State University Raleigh North Carolina USA

**Keywords:** gait kinematics, giraffe skin disease, locomotion, Masai giraffe

## Abstract

Emerging skin diseases have severely impacted wildlife in recent decades, with consequences ranging from increased morbidity and mortality to local extinction and widespread biodiversity loss. Individuals that persist with various skin diseases can have sublethal consequences, including altered behavior and impaired locomotor function. Giraffe skin disease (GSD) is a condition that results in skin lesions of varying severity among different giraffe (*Giraffa* spp.) populations throughout Africa. Prior reports have suggested that individuals with limb lesions from GSD do not have increased mortality but rather suffer from lameness. We examined whether GSD severity and unilateral versus bilateral forelimb lesions differentially impact spatiotemporal gait kinematics and carpus joint angle kinematics of Masai giraffe (*G. tippelskirchi tippelskirchi*) in Ruaha National Park, Tanzania. We found that GSD lesions altered normal walking gait kinematics (i.e., decreased walking speed and increased stride duration) largely irrespective of lesion severity or number of limbs affected. Impaired movement due to GSD could negatively impact foraging efficiency, dispersal, and predator susceptibility. Given that wildlife skin diseases are predicted to become more prevalent with climate change, examinations of their sublethal effects, in addition to their effects on mortality, are required to better understand long‐term ramifications.

## Graphical abstract

We found that giraffe skin disease lesions altered normal walking gait kinematics largely irrespective of lesion severity or number of limbs affected. Impaired movement due to GSD could negatively impact foraging efficiency, dispersal, and/or predator susceptibility; however, additional research is required to explicitly address these topics.

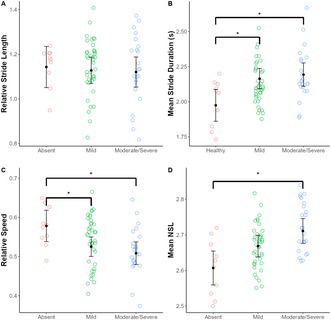

## 1 Introduction

Emerging skin diseases have severely impacted a variety of wildlife in recent decades with consequences ranging from increased morbidity and mortality, extirpation, and extinction. For example, white‐nose syndrome plagues several species of hibernating North American bats (Blehert et al. 2009). Ophidiomycosis (i.e., snake fungal disease) is now observed in dozens of snake species throughout the eastern United States (Lorch et al. 2016). Chytridiomycosis has decimated hundreds of amphibian species worldwide, leading to tremendous biodiversity loss (Kriger and Hero 2009). While these diseases are often fatal and can have devastating effects on populations, individuals that persist with these skin diseases can have sublethal consequences including altered behavior and impaired locomotor function. For example, bats with white‐nose syndrome that survive hibernation often have severe wing damage that is thought to reduce flight performance and foraging efficiency (Reichard and Kunz 2009). Northern leopard frogs ( *Lithobates pipiens* ) suffering from chytridiomycosis were found to have decreased jumping velocities, which may impact their ability to avoid predators (Chatfield et al. 2013). Eastern Massasauga rattlesnakes ( *Sistrurus catenatus* ) with ophidiomycosis moved less frequently compared to unaffected conspecifics (Tetzlaff et al. 2017). Despite the purported effects of these skin diseases, few studies have systematically examined their impacts on locomotor performance and associated downstream ramifications on health and fitness.

Dermatitis and skin lesions have been reported in several terrestrial mammalian orders and have a variety of causative agents including bacteria, fungi, viruses, mites, nematodes, protozoa, and cancer (Ringwaldt et al. 2021). Well‐documented cases include dermatitis and filariosis in rhinoceroses (Mutinda et al. 2012; King'ori et al. 2024), sarcoptic mange affecting many carnivore taxa (Rowe et al. 2019), Tasmanian devil facial tumor disease (McCallum et al. 2009), and lumpy skin disease in domestic cattle and other ruminants (Namazi and Khodakaram Tafti 2021).

Different giraffe skin diseases (GSDs) have emerged in the last 30 years and represent a potential threat to all species of giraffe (*Giraffa* spp.) throughout their range. While GSDs have likely existed prior to this time period, they went largely unnoticed as giraffe were often not a research or management priority. Several etiological agents have been proposed in the pathogenesis of different GSDs, including bacterial, fungal, and nematode origins (Mpanduji et al. 2011; Kiula et al. 2021; Han et al. 2022; Wanda et al. 2025). All GSDs generally manifest as scab‐like lesions; however, the anatomical presentation of the lesions differs by geographical location, with lesions prevalent on the neck, upper body, and/or limbs across different sites (Muneza et al. 2016). GSDs exclusively impact giraffe and are more common in adults (Epaphras et al. 2012; Muneza et al. 2017). To date, no study has systematically assessed the timing of GSD progression; however, published studies differentiate and describe mild, moderate, and severe forms of GSDs (Epaphras et al. 2012; Muneza et al. 2016; 2019). GSDs are widespread throughout Africa, occurring in three giraffe species and in at least 13 parks and reserves across seven countries, including Botswana, Kenya, Namibia, South Africa, Tanzania, Uganda, and Zimbabwe (Muneza et al. 2016).

Bond et al. (2016) conducted a three‐year longitudinal photographic mark and recapture study of Masai giraffe (*G. tippelskirchi tippelskirchi*) in Tarangire National Park (NP) in Tanzania and found no evidence that GSD presence nor lesion severity increased mortality rate; however, others have noted that individuals with GSD lesions on their limbs appeared to move with stiffness and greater difficulty (Epaphras et al. 2012). Understanding whether and how GSD impacts locomotion is critical because impaired movement could negatively impact dispersal and foraging efficiency, and potentially make giraffe more susceptible to predators and poachers. In a recent study of Nubian giraffe ( *G. camelopardalis camelopardalis* ) at Murchison Falls NP (MFNP) in Uganda, Bernstein‐Kurtycz et al. (2023) found that individuals with wire snare injuries had significantly impaired mobility; however, the presence of GSD lesions did not significantly affect locomotor performance. The latter result is unsurprising given that in MFNP lesions were found primarily on the necks of giraffe (Bernstein‐Kurtycz et al. 2023).

The goal of our study is to examine whether and how GSD lesions may impact the locomotion of Masai giraffe at Ruaha NP (RNP), Tanzania—a site where GSD prevalence was estimated at 86% (Muneza et al. 2017) and where GSD lesions are concentrated on the forelimbs (Epaphras et al. 2012; Muneza et al. 2016). We examined whether GSD severity and unilateral versus bilateral forelimb lesions differentially impact spatiotemporal gait kinematics and carpus joint angle kinematics. We predicted that increasing GSD lesion severity and the presence of bilateral lesions will be consistent with impaired locomotor function, including decreased stride length and walking speed. Furthermore, because previous reports indicated that giraffe appeared to have stiffness on limbs affected with GSD lesions (Epaphras et al. 2012), we predicted increasing GSD lesion severity would lead to reduced range of motion at the carpus joint.

## 2 Materials and Methods

### 2.1 Subjects and Video Recordings

**FIGURE 1:**
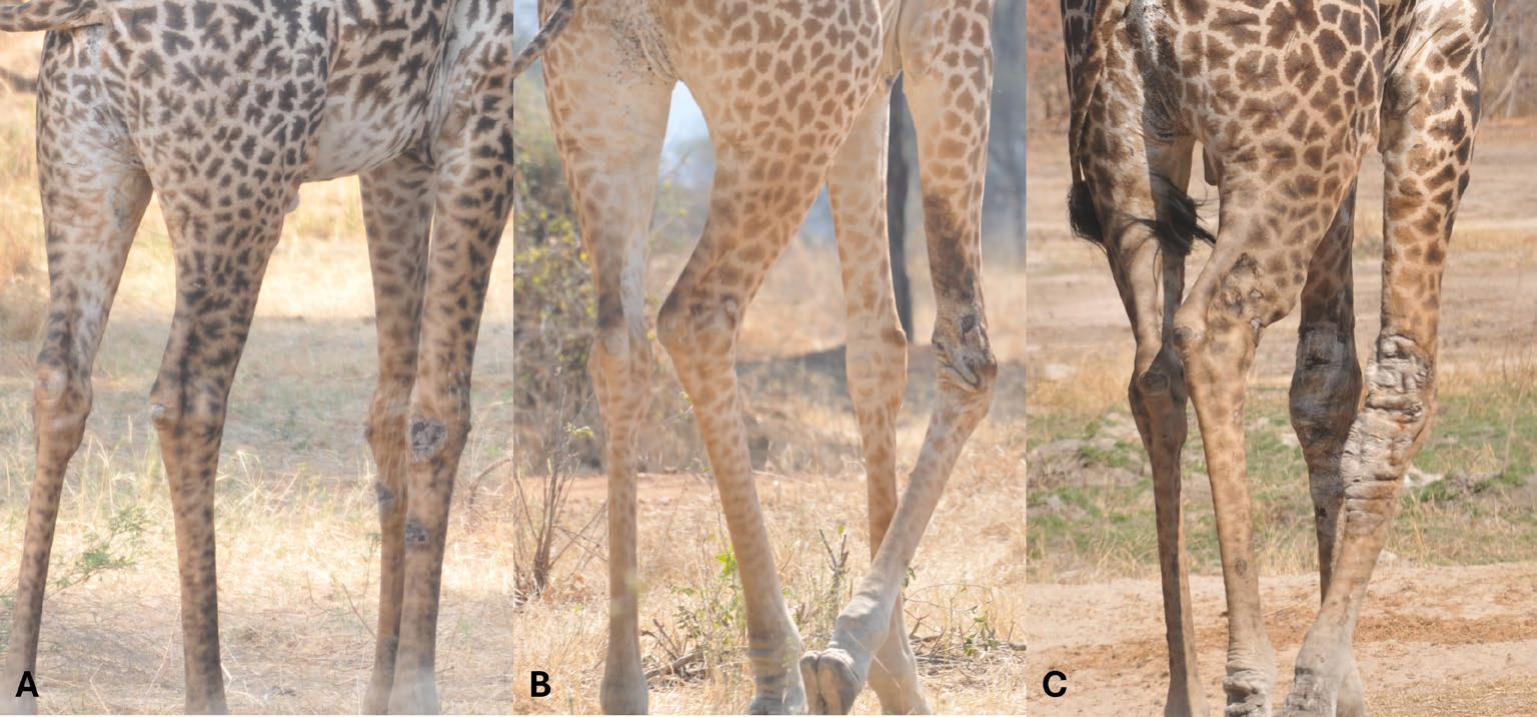
Examples of giraffe skin disease forelimb lesions, including severity categories of mild (A), moderate (B), and severe (C). All images taken by Arthur Muneza in Ruaha National Park, Tanzania.

We opportunistically filmed 40 adult Masai giraffe (*n* = 10 males; *n* = 30 females) in RNP from July 2021 to February 2022. Individuals were identified based on unique and unchanging coat patterns (Foster 1966). All videos were recorded at ~50–150 m from the focal animal and on flat ground to control for potential gait adjustments in response to inclined/declined terrain. Each giraffe was assigned to one of four GSD condition categories: (1) absent (i.e., no visible GSD lesions), (2) mild GSD, (3) moderate GSD, or (4) severe GSD (Figure 1). GSD severity was scored according to Epaphras et al. (2012). We also recorded whether lesions occurred on one or both forelimbs (i.e., unilateral vs. bilateral).

Giraffe locomotion at RNP was filmed at 30 frames per second using a Nikon D7000 DSLR with a 70–200 mm lens. We included only videos with unobstructed views of limb touchdowns (*n* = 36 videos). Videos included in the spatiotemporal gait analyses contained between one and four strides per individual (*n* = 72 strides). We attempted to film perpendicular to the line of travel when possible; however, parallax does not impact the timing and digitizing limb touchdown and liftoff events. All spatial points (i.e., stride length and shoulder height) were digitized in the same video frame for a given video clip, mitigating potential distortion issues due to parallax (Dunham et al. 2018). Angular measurements are susceptible to error from parallax, so only strides in which giraffe were moving approximately perpendicular to the camera were used to quantify carpus joint angle measurements (*n* = 54 strides). We used GaitKeeper, an open‐source MATLAB package, to digitize limb liftoff and touchdown events, shoulder height, and stride length (Dunham et al. 2018) (http://www.younglaboratory.org/GaitKeeper). We recorded neck angle measurements for individual videoframes using the angle tool in ImageJ (Rueden et al. 2017).

### 2.2 Spatiotemporal Limb Kinematics

Our spatiotemporal gait kinematic methods mirror those reported by Bernstein‐Kurtycz et al. 2023. We quantified giraffe shoulder height (in pixels) by digitizing a point at roughly the height of the glenohumeral joint and another point at ground level directly below the shoulder. We quantified stride length (in pixels) by digitizing the initial touchdown of a reference limb (e.g., left hindlimb) and the subsequent reference limb touchdown. Relative stride length was then calculated by dividing stride length in pixels by shoulder height in pixels. Relative (i.e., dimensionless) stride length was reported to account for differences in body size among giraffe. Stride duration was recorded as the duration (in seconds) between the initial and subsequent touchdown of a reference limb. We generated a mean stride duration based on the values for each of the four limbs. To control for potential speed differences due to differences in body size among giraffe, we calculated relative speed by dividing relative stride length by mean stride duration, resulting in values with units of % of shoulder height per second. We quantified the portion of stride duration in which individuals were supported by zero, one, two, three, or four limbs to generate mean number of supporting limbs (i.e., mean NSL) throughout the stride (Shapiro and Young 2012; Bernstein‐Kurtycz et al. 2023).

### 2.3 Carpus Joint Angle Kinematics

Because GSD lesions in RNP giraffe often manifest on the forelimb and specifically on the limb segments at the level of the radioulna and metacarpal (Muneza et al. 2016), we examined angular kinematics at the carpus joint (i.e., the angle between the radioulna and metacarpal). We quantified carpus joint angle during maximum flexion and maximum extension for each stride. Points located at the center of the elbow, carpus, and metacarpophalangeal joint were digitized to generate the carpus joint angle (Basu and Hutchinson 2022). We digitized carpus angle in each video frame using ImageJ (Rueden et al. 2017) and identified peak flexion (i.e., minimum value) and peak extension (i.e., maximum value). We then calculated carpus angle range of motion (ROM) as the difference between peak flexion and peak extension.

### 2.4 Statistical Analyses

We used linear mixed models to examine the effect of GSD lesion severity (i.e., absent, mild, moderate, and severe) on spatiotemporal limb kinematics, including relative stride length, mean stride duration, relative speed, and mean NSL. Similarly, we independently examined the effects of the number of limbs affected with GSD lesions (i.e., absent, unilateral forelimb lesions, and bilateral severe forelimb lesions) on the same spatiotemporal kinematic variables. We also used linear mixed models to assess the effect of GSD severity on carpus joint angle kinematics, including peak flexion, peak extension, and ROM. GSD severity category and number of limbs affected with GSD lesions were included as fixed factors in the different models. For all statistical models, individual giraffes were nested within video clips as a random factor (intercept) to control for random variation between individuals. We included sex as a covariate in all statistical models to control for potential differences in gait kinematics due to differences in body segment proportions between the sexes (Cavener et al. 2023). Relative speed was included as a covariate in the mean NSL models because speed has been shown to negatively correlate with mean NSL in giraffes (Bernstein‐Kurtycz et al. 2023). Relative speed was also included as a covariate in the carpus joint angle models (i.e., peak flexion, peak extension, and ROM) to control for potential speed‐related differences in these variables. Satterthwaite approximations were used to adjust degrees of freedom in cases of heteroscedasticity for all models. Analyses were conducted in R statistical software (R Core Team 2019), including add‐on packages: lme4 and lmerTest (Kuznetsova et al. 2017). We used the emmeans package (Lenth 2025) to generate estimated marginal mean values for each dependent variable listed above. Post hoc pairwise comparisons of mixed models were conducted using the emmeans package, with multiple pairwise comparisons corrected using the false discovery rate method (Benjamini and Hochberg 1995).

## 3 Results

### 3.1 Sample Characteristics

Table 1 displays spatiotemporal limb kinematics and carpus joint angle kinematics sample sizes (i.e., number of strides and individual giraffe) for each GSD severity category. For all statistical analyses, we pooled data for moderate and severe categories due to the small sample size for the latter category (i.e., *n* = 3 individuals). We found that GSD lesions were found exclusively on the forelimbs of giraffe in our sample. Roughly half of the individuals had unilateral forelimb lesions (i.e., 22/42; 52.4%), 31.0% (13/42) had bilateral forelimb lesions, and 16.7% (7/42) had no discernible lesions. For giraffe with GSD lesions, forelimb segments affected included the radioulna (23/35; 65.7%), carpus joint (30/35; 85.7%), and metacarpal (6/35; 17.1%). More than half of the affected giraffe had GSD lesions on multiple limb regions (20/35; 57.1%) (Table 2).

**TABLE 1:** Number of strides used in spatiotemporal kinematics analyses and carpus joint angle analyses for different GSD severity categories in free‐ranging Masai giraffe from Ruaha National Park, Tanzania.

GSD severity	Females (*n* = 30)		Males (*n* = 10)	
	Spatiotemporal kinematics strides	Carpus joint angle strides	Spatiotemporal kinematics strides	Carpus joint angle strides
Absent	5	3	5	5
Mild	33	25	6	5
Moderate	13	9	3	2
Severe	3	3	4	2
Total	54	40	18	14

**TABLE 2:** Number of forelimbs affected with GSD lesions in free‐ranging Masai giraffe sample from Ruaha National Park, Tanzania.

Forelimbs affected	Females (*n* = 30)	Males (*n* = 10)
	Number of strides	Number of strides
Absent	5	5
Unilateral	26	5
Bilateral	23	8
Total	54	18

### 3.2 Effect of GSD Severity on Spatiotemporal Limb Kinematics

**FIGURE 2:**
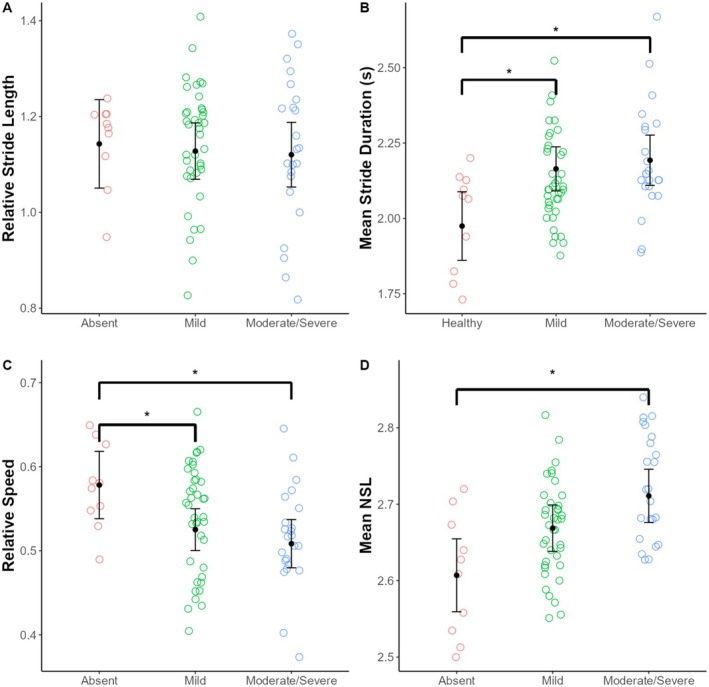
Variation in spatiotemporal limb kinematics among GSD forelimb lesion severity categories (i.e., absent, mild, and moderate/severe) in free‐ranging Masai giraffe from Ruaha National Park, Tanzania. Each point represents a stride. Black dots indicate estimated marginal means and black error bars indicate 95% confidence intervals from the linear mixed models. NSL, number of supporting limbs.

Giraffe skin disease severity had a significant effect on three of the spatiotemporal kinematic variables examined (Table 3). Individuals without GSD lesions (i.e., absent) had significantly shorter relative stride durations compared to individuals with mild lesions and those with moderate/severe lesions (*p* = 0.01 for both comparisons). Similarly, individuals without GSD lesions had greater relative speed compared to individuals with mild lesions and those with moderate/severe lesions (*p* = 0.04 and *p* = 0.02, respectively). Lastly, individuals with moderate/severe GSD lesions had significantly greater mean NSL values compared to individuals without lesions (*p* = 0.03) (Table 4; Table S1; Figure 2).

**TABLE 3:** Effects of GSD severity on spatiotemporal limb kinematics in free‐ranging Masai giraffe from Ruaha National Park, Tanzania.

Parameter	*F*	df	*p*
Relative stride length	0.08	2, 41.1	0.92
Mean stride duration	5.19	2, 39.6	0.01
Relative speed	4.22	2, 37.9	0.02
Mean NSL	4.00	2, 40.3	0.03

Abbreviation: NSL, number of supporting limbs.

**TABLE 4:** Pairwise comparisons of estimated marginal means for spatiotemporal limb kinematics among different GSD severity conditions in free‐ranging Masai giraffe. Multiple comparisons were corrected using the false discovery rate method. Parentheses contain 95% confidence intervals. Shared superscripts within a row indicate that estimated marginal means were not significantly different.

Parameter	Absent	Mild	Moderate/Severe
Relative stride length	1.14^a^ (1.05–1.24)	1.13^a^ (1.07–1.19)	1.12^a^ (1.05–1.19)
Mean stride duration (s)	1.97^a^ (1.86–2.09)	2.16^b^ (2.09–2.24)	2.19^b^ (2.11–2.28)
Relative speed	0.58^a^ (0.54–0.62)	0.53^b^ (0.50–0.55)	0.51^b^ (0.48–0.54)
Mean NSL	2.62^a^ (2.57–2.67)	2.66^ab^ (2.63–2.69)	2.70^b^ (2.67–2.74)

Abbreviation: NSL, number of supporting limbs.

### 3.3 Effect of Unilateral Versus Bilateral Lesions on Spatiotemporal Limb Kinematics

**FIGURE 3:**
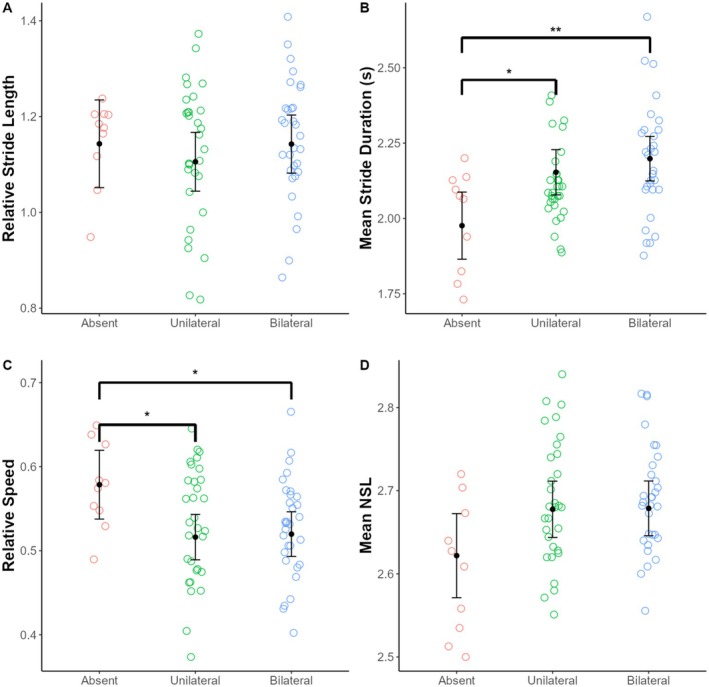
Variation in spatiotemporal limb kinematics among Masai giraffe without GSD lesions (i.e., absent) and those with unilateral and bilateral GSD forelimb lesions in Ruaha National Park, Tanzania. Each point represents a stride. Black dots indicate estimated marginal means and black error bars indicate 95% confidence intervals from the linear mixed models. NSL, number of supporting limbs.

The number of forelimbs affected with GSD lesions (i.e., absent, unilateral, or bilateral lesions) had a significant effect on two of the three spatiotemporal kinematic variables examined (Table 5). Individuals without GSD lesions had significantly shorter relative stride durations compared to individuals with unilateral forelimb lesions and those with bilateral forelimb lesions (*p* = 0.02 and *p* = 0.006, respectively). Individuals without lesions exhibited significantly faster relative speeds compared to individuals with unilateral lesions and those with bilateral lesions (*p* = 0.03 for both comparisons) (Table 6; Table S2; Figure 3).

**TABLE 5:** Effects of unilateral versus bilateral GSD lesions on spatiotemporal limb kinematics in free‐ranging Masai giraffe from Ruaha National Park, Tanzania.

Parameter	*F*	df	*p*
Relative stride length	0.57	2, 42.8	0.57
Mean stride duration	5.68	2, 39.5	0.007
Relative speed	3.63	2, 39.3	0.04
Mean NSL	1.92	2, 45.6	0.16

Abbreviation: NSL, number of supporting limbs.

**TABLE 6:** Pairwise comparisons of estimated marginal means for spatiotemporal limb kinematics for unilateral versus bilateral GSD lesions in free‐ranging Masai giraffe. Multiple comparisons were corrected using the false discovery rate method. Parentheses contain 95% confidence intervals. Shared superscripts within a row indicate that estimated marginal means were not significantly different.

Parameter	Absent	Unilateral	Bilateral
Relative stride length	1.14^a^ (1.05–1.24)	1.11^a^ (1.04–1.17)	1.14^a^ (1.08–1.20)
Mean stride duration (s)	1.97^a^ (1.86–2.09)	2.15^b^ (2.08–2.23)	2.20^b^ (2.12–2.27)
Relative speed	0.58^a^ (0.54–0.62)	0.52^b^ (0.49–0.54)	0.52^b^ (0.49–0.55)
Mean NSL	2.62^a^ (2.57–2.67)	2.68^a^ (2.64–2.71)	2.68^a^ (2.65–2.71)

Abbreviation: NSL, number of supporting limbs.

### 3.4 Effect of GSD Severity on Carpus Joint Angle Kinematics

**FIGURE 4:**
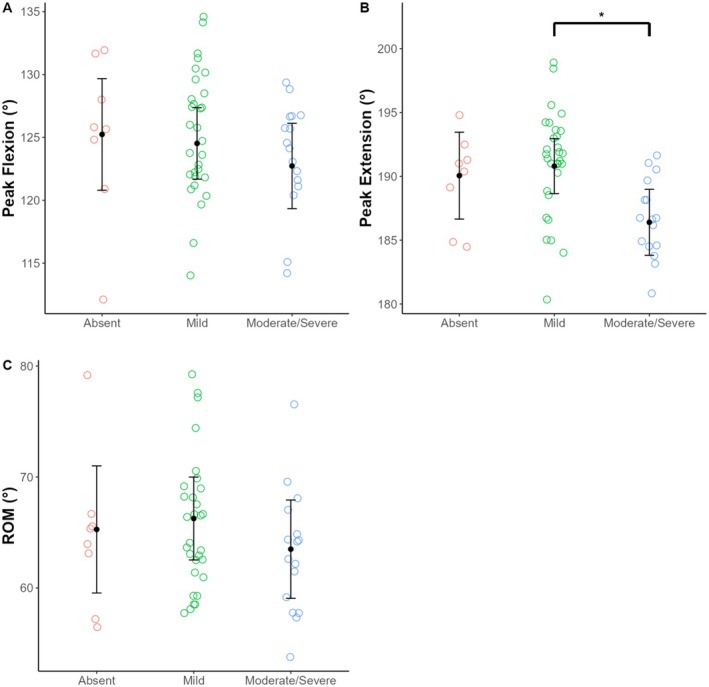
Variation in carpus joint angle kinematics among GSD forelimb lesion severity categories (i.e., absent, mild, and moderate/severe) in free‐ranging Masai giraffe from Ruaha National Park, Tanzania. Each point represents a stride. Black dots indicate estimated marginal means and black error bars indicate 95% confidence intervals from the linear mixed models. ROM, range of motion.

Giraffe skin disease severity had a significant effect on carpus joint angle peak extension (Table 7). Peak extension was significantly greater in individuals with mild lesions compared to those with moderate/severe lesions (*p* = 0.02) (Table 8; Table S3; Figure 4). All other comparisons of carpus joint angle kinematics were not significant.

**TABLE 7:** Effects of GSD severity on carpus joint angle kinematics in free‐ranging Masai giraffe from Ruaha National Park, Tanzania.

Parameter	*F*	df	*p*
Peak flexion	0.59	2, 17.1	0.56
Peak extension	4.69	2, 21.2	0.02
ROM	0.60	2, 19.5	0.56

Abbreviation: ROM, range of motion.

**TABLE 8:** Pairwise comparisons of estimated marginal means for carpus joint angle kinematics in free‐ranging Masai giraffe. Multiple comparisons were corrected using the false discovery rate method. Parentheses contain 95% confidence intervals. Shared superscripts within a row indicate that estimated marginal means were not significantly different.

Parameter	Absent	Mild	Moderate/Severe
Peak flexion	125.2^a^ (120.8–129.7)	124.5^a^ (121.7–127.4)	122.7^a^ (119.3–126.1)
Peak extension	190.1^ab^ (186.6–193.5)	190.8^a^ (188.6–193.0)	186.4^b^ (183.8–189.0)
ROM	65.3^a^ (59.5–71.0)	66.3^a^ (62.5–70.0)	63.5^a^ (59.1–67.9)

Abbreviation: ROM, range of motion.

## 4 Discussion

We found that GSD lesions significantly impacted the walking gait kinematics of Masai giraffe in RNP. Importantly, some kinematic differences manifested regardless of GSD severity or whether lesions affected one or both forelimbs. That is, even mild lesions on one forelimb significantly impacted at least some gait kinematic parameters. Most notably, the average walking speed of individuals without discernible lesions was ~9% faster than individuals with mild lesions and ~12% faster than individuals with moderate/severe lesions. The differences in walking speed were attributed to increased stride durations (i.e., decreased stride frequency) of giraffe with GSD lesions. Increasing stride duration can be accomplished by prolonging the stance phase of the gait cycle and/or by slowing the swing phase. We found support for the former in our dataset as evidenced by the increased mean NSL in individuals with moderate/severe lesions compared to individuals without lesions. This suggests giraffe affected with GSD prioritized increasing ground contact time throughout the stride, potentially as a strategy to reduce loading on the affected limb by dissipating forces across a greater number of limbs. While not statistically significant, our results also generally show that individuals with moderate/severe lesions had reduced walking speeds and greater mean NSL compared to individuals with mild lesions. The lack of statistical significance could be due to the small sample size for individuals with moderate or severe lesions.

Our findings are contrary to our prediction and the results of previous studies that found giraffes modulated walking speed by adjusting stride length while maintaining consistent stride durations (Basu et al. 2019). Furthermore, Bernstein‐Kurtycz et al. (2023) found that Nubian giraffes suffering from snare wounds and characterized by significantly slower walking speeds (i.e., ~14% slower than individuals without GSD lesions) were found to maintain normal stride durations while adopting shorter stride lengths. Taken together, these results illustrate that snare wounds and forelimb GSD lesions reduce giraffe walking speed to similar degrees, albeit in different ways.

Contrary to our predictions, GSD severity largely did not impact carpus joint angle kinematics. Previous reports indicated that Masai giraffe appeared to have stiffness on limbs affected with GSD lesions (Epaphras et al. 2012), which we predicted would manifest as decreased ROM at the affected carpus joint. Roughly 85% of the affected Masai giraffe in our sample had lesions at the level of the carpus joint. While ROM at the carpus joint was lowest for individuals with moderate/severe GSD lesions in our sample, this result was not statistically significant. Bernstein‐Kurtycz et al. (2023) found that Nubian giraffe affected with GSD lesions on their necks also maintained consistent neck ROM compared to individuals without GSD lesions. While it is not yet clear why GSD lesions manifest at different anatomical locations (e.g., neck vs. forelimb) across different sites (Muneza et al. 2016), our results, combined with those from MFNP, suggest that GSD lesions do not significantly constrain joint mobility at the affected areas.

Impaired mobility of individuals with GSD lesions could have important consequences for giraffe movement ecology. Adult female Masai giraffe (i.e., including 18 individuals sampled from Serengeti and Tarangire NPs in Tanzania, and Amboseli NP in Kenya) traveled ~12.1 km per day and had a mean home range size of 205.2 km^2^ (Brown et al. 2023). Future research is required to assess whether individuals with GSD have shorter daily travel distances or reduced home range sizes compared to unaffected individuals. Reduced mobility and potential flight ability may also make affected individuals more vulnerable to predators. Giraffe are a preferred food resource for African lion ( *Panthera leo* ) in RNP (Muneza et al. 2022). Lion may identify the external symptoms of giraffe with GSD (e.g., visible skin lesions and impaired mobility) and preferentially target individuals with GSD. Muneza et al. (2022) documented a positive relationship between severe GSD lesions and signs of attempted lion predation, including bite marks, claw marks, and missing tails at RNP. However, because the authors found no evidence that GSD severity impacted the likelihood of surviving a lion attack, there is currently no direct evidence that it increases lion predation susceptibility. Furthermore, because our study was limited to walking gaits, future research is required to determine if GSD lesions adversely impact running gaits and maneuverability, which are more direct measures of an individual's ability to escape from predators.

## 5 Conclusion

Emerging skin diseases have had devastating effects on wildlife populations in recent decades, and new zoonotic threats are likely to emerge in conjunction with continuing climate change (Garcia‐Solache and Casadevall 2010; Fisher et al. 2012). Published studies indicate that some mammals can recover from skin diseases with appropriate interventions: filariosis in white and black rhinoceros (Mutinda et al. 2012), sarcoptic mange in a variety of mammalian taxa (Rowe et al. 2019), and cutaneous enzootic myiasis in samba deer (Pereira et al. 2020). Conversely, some skin diseases are characterized by high mortality rates, including Tasmanian devil facial tumor disease (McCallum et al. 2009) and white‐nose syndrome in North American bats (Blehert et al. 2009). Current evidence suggests that GSD does not measurably increase mortality (Bond et al. 2016), and preliminary interventions have been unsuccessful in eliminating GSD (TAWIRI 2023). We showed that GSD had sublethal effects on locomotion, most notably with affected individuals walking significantly slower compared to individuals without GSD lesions. Giraffe may adapt to these morbidity impacts of GSD, but these kinematic discrepancies may also have downstream consequences for giraffe movement ecology. Future research should continue to examine sublethal effects of emerging skin diseases on wildlife to better understand their long‐term ramifications.

## Author Contributions

**N. T. Dunham:** conceptualization (equal), formal analysis (lead), methodology (lead), writing – original draft (lead). **L. M. Bernstein‐Kurtycz:** conceptualization (equal), methodology (equal), writing – review and editing (equal). **J. Manzak:** conceptualization (equal), investigation (equal), writing – review and editing (equal). **A. B. Muneza:** conceptualization (equal), methodology (equal), writing – review and editing (equal). **M. B. Brown:** conceptualization (equal), investigation (equal), methodology (equal), writing – review and editing (equal). **J. Fennessy:** conceptualization (equal), investigation (equal), writing – review and editing (equal). **P. M. Dennis:** conceptualization (equal), writing – review and editing (equal). **C. J. Kendall:** conceptualization (equal), writing – review and editing (equal). **K. E. Lukas:** conceptualization (equal), writing – review and editing (equal).

## Conflicts of Interest

The authors declare no conflicts of interest.

## Supporting information


Tables S1–S3.

## Data Availability

Data used in the analyses are available via Dryad: https://doi.org/10.5061/dryad.qbzkh18vg.
