# Supplementary material for: Exploring the Effects of Giraffe Skin Disease Limb Lesions on Locomotion
Source: Ecol Evol. 2025 Jul 10;15(7):e71774. doi: 10.1002/ece3.71774 (PMC12245478; doi:10.1002/ece3.71774)
Supplement: Supplementary file 1 — Tables S1–S3. [file ECE3-15-e71774-s001.docx]

Table S1. Posthoc pairwise comparisons of estimated marginal means for spatiotemporal limb kinematics among different GSD severity conditions in free-ranging Masai giraffe. Comparisons were conducted using the emmeans package, with multiple pairwise comparisons corrected using the false discovery rate method.

| Contrast | p-value |
| --- | --- |
| **Relative Stride Length** |  |
| Absent - Mild | 0.85 |
| Absent – Moderate/Severe | 0.85 |
| Mild – Moderate/Severe | 0.85 |
|  |  |
| **Mean Stride Duration** |  |
| Absent - Mild | 0.01 |
| Absent – Moderate/Severe | 0.01 |
| Mild – Moderate/Severe | 0.57 |
|  |  |
| **Relative Speed** |  |
| Absent - Mild | 0.04 |
| Absent – Moderate/Severe | 0.02 |
| Mild – Moderate/Severe | 0.34 |
|  |  |
| **Mean NSL** |  |
| Absent - Mild | 0.05 |
| Absent – Moderate/Severe | 0.003 |
| Mild – Moderate/Severe | 0.05 |

NSL = number of supporting limbs

Table S2. Posthoc pairwise comparisons of estimated marginal means for spatiotemporal limb kinematics for unilateral vs. bilateral GSD lesions in free-ranging Masai giraffe. Comparisons were conducted using the emmeans package, with multiple pairwise comparisons corrected using the false discovery rate method.

| Contrast | p-value |
| --- | --- |
| **Relative Stride Length** |  |
| Absent - Unilateral | 0.75 |
| Absent – Bilateral | 0.99 |
| Unilateral – Bilateral | 0.75 |
|  |  |
| **Mean Stride Duration** |  |
| Absent - Unilateral | 0.02 |
| Absent – Bilateral | 0.006 |
| Unilateral – Bilateral | 0.33 |
|  |  |
| **Relative Speed** |  |
| Absent - Unilateral | 0.03 |
| Absent – Bilateral | 0.03 |
| Unilateral – Bilateral | 0.83 |
|  |  |
| **Mean NSL** |  |
| Absent - Unilateral | 0.12 |
| Absent – Bilateral | 0.12 |
| Unilateral – Bilateral | 0.95 |

NSL = number of supporting limbs

Table S3. Posthoc pairwise comparisons of estimated marginal means for carpus joint angle kinematics among different GSD severity conditions in free-ranging Masai giraffe. Comparisons were conducted using the emmeans package, with multiple pairwise comparisons corrected using the false discovery rate method.

| Contrast | p-value |
| --- | --- |
| **Peak Flexion** |  |
| Absent - Mild | 0.79 |
| Absent – Moderate/Severe | 0.58 |
| Mild – Moderate/Severe | 0.58 |
|  |  |
| **Peak Extension** |  |
| Absent - Mild | 0.72 |
| Absent – Moderate/Severe | 0.16 |
| Mild – Moderate/Severe | 0.02 |
|  |  |
| **ROM** |  |
| Absent - Mild | 0.78 |
| Absent – Moderate/Severe | 0.78 |
| Mild – Moderate/Severe | 0.78 |

ROM = range of motion
